# Introducing a new estimator and test for the weighted all-cause hazard ratio

**DOI:** 10.1186/s12874-019-0765-1

**Published:** 2019-06-11

**Authors:** Ann-Kathrin Ozga, Geraldine Rauch

**Affiliations:** 1 Institute of Medical Biometry and Epidemiology, University Medical Center Hamburg-Eppendorf, Martinistraße 52, Hamburg, 20246 Germany; 2 Charité - Universitätsmedizin Berlin, corporate member of Freie Universität Berlin, Humboldt-Universität zu Berlin, Institute of Biometry and Clinical Epidemiology, Charitéplatz 1, Berlin, 10117 Germany; 3 Berlin Institute of Health (BIH), Anna-Louisa-Karsch 2, Berlin, 10178 Germany

**Keywords:** Composite endpoint, Weighted effect measure, Weight-based log-rank test, Simulation study

## Abstract

**Background:**

The rationale for the use of composite time-to-event endpoints is to increase the number of expected events and thereby the power by combining several event types of clinical interest. The all-cause hazard ratio is the standard effect measure for composite endpoints where the all-cause hazard function is given as the sum of the event-specific hazards. However, the effect of the individual components might differ, in magnitude or even in direction, which leads to interpretation difficulties. Moreover, the individual event types often are of different clinical relevance which further complicates interpretation. Our working group recently proposed a new weighted effect measure for composite endpoints called the ‘weighted all-cause hazard ratio’. By imposing relevance weights for the components, the interpretation of the composite effect becomes more ‘natural’. Although the weighted all-cause hazard ratio seems an elegant solution to overcome interpretation problems, the originally published approach has several shortcomings: First, the proposed point estimator requires pre-specification of a parametric survival model. Second, no closed formula for a corresponding test statistic was provided. Instead, a permutation test was proposed. Third, no clear guidance for the choice of the relevance weights was provided. In this work, we will overcome these problems.

**Methods:**

Within this work a new non-parametric estimator and a related closed formula test statistic are presented. Performance of the new estimator and test is compared to the original ones by a Monte-Carlo simulation study.

**Results:**

The original parametric estimator is sensible to miss-specifications of the survival model. The new non-parametric estimator turns out to be very robust even if the required assumptions are not met. The new test shows considerably better power properties than the permutation test, is computationally much less expensive but might not preserve type one error in all situations. A scheme for choosing the relevance weights in the planning stage is provided.

**Conclusion:**

We recommend to use the non-parametric estimator along with the new test to assess the weighted all-cause hazard ratio. Concrete guidance for the choice of the relevance weights is now available. Thus, applying the weighted all-cause hazard ratio in clinical applications is both - feasible and recommended.

**Electronic supplementary material:**

The online version of this article (10.1186/s12874-019-0765-1) contains supplementary material, which is available to authorized users.

## Background

In many clinical trials, the aim is to compare two treatment groups with respect to a rarely occurring event like myocardial infarction or death. In this situation, a high number of patients has to be included and observed over a long period of time for a demonstration of a relevant treatment effect and to reach an acceptable power. Combining several events of interest within a so-called composite endpoint can lead to a smaller required sample size and save time as a higher number of events is mend to increase the power. The common treatment effect measure for composite endpoints is the all-cause hazard ratio. This effect measure is based on the total number of events irrespective of their type. Commonly, either the log-rank test or the Cox proportional hazards model [1–4] are used for analysing the all-cause hazard ratio. However, the interpretation of the all-cause hazard ratio as a composite treatment effect can be difficult. This is due to two reasons: First, the composite might not necessarily reflect the effects of the individual components which can differ in magnitude or even in direction [5–7]. Second, the distinct event types could be of different clinical relevance. For example, the fatal event ‘death’ is more relevant than a non-fatal event like ‘cardiovascular hospital admission’. Moreover, the less relevant event often contributes a higher number of events and therefore has a higher influence on the composite effect than the less relevant event.

Current guidelines on clinical trial methodology hence recommend to combine only events of the same clinical relevance [3, 8]. However, this is rather unrealistic in clinical practice, as important components like ‘death’ cannot be excluded from the primary analysis if a fatal event is clearly more relevant than any other non-fatal event. Therefore, to address the problems that arise within the analysis of a composite endpoint other methods to ease the interpretation of results are needed. An intuitive approach could be to define a weighted composite effect measure with weights that reflect the different levels of clinical relevance of the components. Weighted effect measures have been proposed and compared by several authors [9–14]. Some of the main disadvantages of these approaches include the high dependence on the censoring mechanism and on competing risks [13, 14]. Recently, Rauch et al. [15] proposed a new weighted effect measure called the ‘weighted all-cause hazard ratio’. This new effect measure is defined as the ratio between the weighted average of the cause-specific hazards for two groups. Thereby, the predefined weights are assigned to the individual cause-specific hazards. With equal weights for the components the weighted all-cause hazard ratio corresponds to the common all-cause hazard ratio and thus defines a natural extension of the standard approach.

Although this new weighted effect measure seems an elegant solution to overcome interpretation problems, the originally published approach has several shortcomings: 1. The proposed original estimator for the weighted all-cause hazard ratio requires pre-specification of a parametric survival model to estimate the individual cause-specific hazards. The form of the survival model, however, is usually not known in the planning stage of a trial. 2. No closed formula for a corresponding test statistic was introduced but a permutation test was used instead which comes along with a high computational effort. 3. No clear guidance for the choice of the relevance weighting factors was provided. In this work, we want to address these issues to make the weighted all-cause hazard ratio more appealing for practical application. In particular we will provide answers to the following questions:

- How robust is the original estimator for the weighted all-cause hazard ratio against miss-specifications of the underlying parametric survival model?
- How robust is the new alternative non-parametric estimator for the weighted all-cause hazard ratio?
- How can we derive a closed formula test statistic for testing the weighted all-cause hazard ratio?
- How do the different estimators and tests behave in a direct performance comparison?
- What are the required steps when choosing adequate weighting factors in the planning stage?

This paper is organized as follows: In the Methods Section, we start by introducing the standard unweighted approach for analysing a composite time-to-first event endpoint. In the same section, the weighted all-cause hazard ratio is introduced as well as the original parametric estimator and the permutation test as recently proposed by Rauch et al. [15]. A new non-parametric estimator for the weighted all-cause hazard ratio and a related closed formula test is introduced subsequently. Next, we provide a step-by-step guidance on the choice of the relevance weighting factors. In the Results Section, the different estimators and tests for the weighted all-cause hazard ratio are compared by means of a Monte-Carlo simulation study to evaluate their performance for various data scenarios, in particular those who meet and those who violate the underlying model assumptions. We discuss our methods and results and we finish the article with concluding remarks.

## Methods

### The standard all-cause hazard ratio

The interest lies, throughout this work, in a two-arm clinical trial where an intervention *I* shall be compared to a control *C* with respect to a composite time-to-event endpoint. A total of *n* individuals are randomized in a 1:1 allocation to the two groups. The composite endpoint consists of *k* components *EP*_*j*_, *j*=1,...,*k*. It is assumed that a lower number of events corresponds to a more favourable result. The observational period is given by the interval [0,*τ*]. The study aim is to demonstrate superiority of the new intervention and therefore a one-sided test problem is formulated.

#### Definitions and test problem

The all-cause hazard function for the composite endpoint is parametrized as

$$\begin{array}{*{20}l} &\lambda_{CE,i}(t)=\lambda_{CE,0}(t)\exp(\beta_{CE}X_{i}),\\ & i=1,...,n,\ \end{array} $$

where *X*_*i*_ is the treatment indicator which equals 1 when the individual *i* belongs to the intervention group and 0 when it belongs to the control. Equivalently, the cause-specific hazards for the components are given as

$$\begin{array}{*{20}l} &\lambda_{EP_{j},i}(t)=\lambda_{EP_{j},0}(t)\exp(\beta_{EP_{j}}X_{i}),\\ & i=1,...,n,\ j=1,...,k. \end{array} $$

Note that the hazard for the composite endpoint is the sum of the cause-specific hazards for the components [15]

1 $$\begin{array}{@{}rcl@{}} \lambda_{CE}(t)=\sum_{j=1}^{k}{\lambda_{EP_{j}}(t)}. \end{array} $$

The all-cause hazard ratio for the composite is given as

$$\begin{array}{@{}rcl@{}} \theta_{CE}=\exp(\beta_{CE})=\frac{\lambda^{I}_{CE}(t)}{\lambda^{C}_{CE}(t)},  \end{array} $$

where the indices *I* and *C* denote the group allocation and proportional hazards are assumed so that *θ*_*CE*_ is constant in time. Note that the proportional hazards assumption can only hold true for both the composite *and* for the components if equal cause-specific baseline hazards are assumed across all components.

As motivated above, a one-sided test problem for the all-cause hazard ratio is considered. The hypotheses thus read as

2 $$\begin{array}{@{}rcl@{}} H_{0}:\theta_{CE}\geq 1 \quad \textnormal{versus} \quad H_{1}: \theta_{CE} <1.  \end{array} $$

#### Point estimator and test statistic

For estimating the all-cause hazard ratio, a semi-parametric estimator for the all-cause hazard ratio $\widehat {\theta }_{CE}$ can be obtained by means of partial maximum-likelihood estimator from the well-known Cox-model [1].

The most common statistical test to assess the null hypothesis stated in (2) is the log-rank test. Let *t*_*l*_, *l*=1,...,*d*, denote the distinct ordered event times for the pooled sample of both groups, where *d* is the total number of observed events irrespective of its type within the observational period [0,*τ*]. Moreover, let $d_{EP_{j},l}=d_{EP_{j},l}^{I}+d_{EP_{j},l}^{C},\ l=1,...,d,\ j=1,...,k$ denote the observed amount of individuals that experience an event of type *j* at time *t*_*l*_ in the pooled sample given as the sum of the specific group-wise number of events. Similarly, let $d_{l}=d_{l}^{I}+d_{l}^{C}=\sum \limits _{j=1}^{k}d_{EP_{j},l}^{I}+\sum \limits _{j=1}^{k}d_{EP_{j},l}^{C},$ denote the observed amount of individuals that experience an event of any type until time *t*_*l*_ in the pooled sample given as the sum of the group-wise number of events. The number of individuals at risk just before time *t*_*l*_ is denoted as $n_{l}=n_{l}^{I}+n_{l}^{C}$. The Nelson-Aalen estimators for the cumulative all-cause hazard functions over the entire observational period are given as

$$\begin{array}{@{}rcl@{}} \hat\Lambda_{CE}^{I}(\tau)=\sum_{t_{l}\leq\tau}\frac{\sum\limits_{j=1}^{k}{d^{I}_{EP_{j},l}}}{n_{l}^{I}}=\sum_{t_{l}\leq\tau}\frac{{d^{I}_{l}}}{n_{l}^{I}} \end{array} $$

and

$$\begin{array}{@{}rcl@{}} \hat\Lambda_{CE}^{C}(\tau)=\sum_{t_{l}\leq \tau}\frac{\sum\limits_{j=1}^{k}{d^{C}_{EP_{j},l}}}{n_{l}^{C}}=\sum_{t_{l}\leq \tau}\frac{{d^{C}_{l}}}{n_{l}^{C}}. \end{array} $$

Under the null hypothesis stated in (2), the cumulative all-cause hazards of both groups are equivalent. This means that the sum of the cause-specific hazards are assumed to be equivalent. This does not automatically imply that the cause-specific hazards are also equivalent. However, this more specific assumption is required to deduce the test statistic of the weight-based log-rank test. Under the null hypothesis (2) and the additional assumption that the cause-specific hazards are equivalent, the random variable $D^{I}_{l},\ l=1,...,d^{I}$, for randomly sampling $d_{l}^{I}$ events from $n_{l}^{I}$ patients where $n_{l}^{I}$ is a subset of the pooled sample with $n_{l}^{I}+n_{l}^{C}$ individuals including a total of *d*_*l*_ events at a fixed time point *t*_*l*_ is hypergeometrically distributed as

$$\begin{array}{@{}rcl@{}} D^{I}_{l}\sim Hyp\left(n_{l}^{I}+n_{l}^{C},d_{l}, d_{l}^{I}\right). \end{array} $$

Then the expectation of the additive $D^{I}_{l}$ over all distinct *t*_*l*_≤*τ* is

$$\begin{array}{@{}rcl@{}} \mathbb{E}\left(\sum_{t_{l}\leq \tau}D^{I}_{l}\right)=\sum_{t_{l}\leq \tau}\mathbb{E}\left(D^{I}_{l}\right) =\sum_{t_{l}\leq \tau}\frac{n_{l}^{I}}{n_{l}^{I}+n_{l}^{C}}d_{l}. \end{array} $$

and the variance is given as

$$\begin{array}{*{20}l} &Var\left(\sum_{t_{l}\leq \tau}D^{I}_{l}\right) =\sum_{t_{l}\leq \tau}Var\left(D_{l}^{I}\right)\\ &=\sum_{t_{l}\leq \tau}{\frac{n_{l}^{I} n_{l}^{C} (n_{l}^{I}+n_{l}^{C}- d_{l}) d_{l}}{(n_{l}^{I}+n_{l}^{C})^{2}(n_{l}^{I}+n_{l}^{C}-1)}}. \end{array} $$

The corresponding log-rank test thus reads as [16]

3 $$\begin{array}{@{}rcl@{}} LR:= \frac{\sum\limits_{t_{l}\leq\tau}{\left(d_{l}^{I}-\frac{n_{l}^{I} d_{l}}{n_{l}^{I}+n_{l}^{C}}\right)}}{\sqrt{\sum\limits_{t_{l}\leq\tau} {\frac{n_{l}^{I} n_{l}^{C} (n_{l}^{I}+n_{l}^{C}- d_{l}) d_{l}}{(n_{l}^{I}+n_{l}^{C})^{2}(n_{l}^{I}+n_{l}^{C}-1)}}}}.  \end{array} $$

The test statistic *LR* is approximately standard normally distributed under the null hypothesis given in (2). Negative values of the test statistic favour the intervention and therefore the null hypothesis is rejected if *LR*≤−*z*_1−*α*_, where *z*_1−*α*_ is the corresponding (1−*α*)-quantile of the standard normal distribution and *α* is the one-sided significance level.

### The weighted all-cause hazard ratio

#### Definitions and test problem

The idea of the weighted all-cause hazard ratio is to replace the standard all-cause hazard given in (1) by a weighted sum of the cause-specific hazards using predefined relevance weights for the individual components that refer to their clinical relevance. The weighted all-cause hazard is then given as

4 $$\begin{array}{@{}rcl@{}} \lambda^{w}_{CE}(t):=\sum_{j=1}^{k}{w_{EP_{j}}\cdot \lambda_{EP_{j}}(t)}  \end{array} $$

where the non-negative weights $w_{EP_{j}}\geq 0$, *j*=1,...,*k*, are reflecting the clinical relevance of the components *EP*_*j*_, *j*=1,...,*k*. If the weights are all equally set to 1 $(w_{EP_{1}}=w_{EP_{2}}=...=w_{EP_{k}}=1)$, then the weighted all-cause hazard corresponds to the standard all-cause hazard.

The ‘weighted all-cause hazard ratio’ as proposed by Rauch et al. [15] is then given as

5 $$\begin{array}{@{}rcl@{}} \theta^{w}_{CE}(t):=\frac{\lambda^{I,w}_{CE}(t)}{\lambda^{C,w}_{CE}(t)},  \end{array} $$

where the indices *I* and *C* denote the group allocation. Note that the weighted all-cause hazard ratio is a time-dependent effect measure except for the case of equal baseline hazards across the components [15] which refers to

$$\begin{array}{@{}rcl@{}} \lambda_{CE,0}(t)=\lambda_{EP_{1},0}(t)=...=\lambda_{EP_{k},0}(t). \end{array} $$

The weighted all-cause hazard ratio can also be integrated over the complete observational period [0,*τ*]

6 $$\begin{array}{@{}rcl@{}} \Theta^{w}_{CE}(\tau):=\frac{1}{\tau}\int_{0}^{\tau}{\theta^{w}_{CE}(t) {\mathrm{d}}t}. \end{array} $$

In the remainder of the work, we will concentrate on the weighted all-cause hazard ratio at a predefined time-point for the sake of simplicity. Again, a one-sided test problem for the weighted all-cause hazard ratio is considered

7 $$\begin{array}{@{}rcl@{}} H_{0}:\theta^{w}_{CE}\geq 1 \quad \textnormal{versus} \quad H_{1}: \theta^{w}_{CE} <1. \end{array} $$

The hypotheses to be assessed in the confirmatory analysis are thus equivalent to the common unweighted approach.

#### Original point estimator and test statistic

In order to estimate the weighted all-cause hazard ratio Rauch et al. [15] proposed to identify and estimate the cause-specific hazards via a parametric survival model. Rauch et al. [15] thereby focused on the Weibull model. This approach is thus based on the assumption that the cause-specific hazards for each component are proportional. With the estimated cause-specific hazards $\hat \lambda ^{I}_{EP_{j}}$ and $\hat \lambda ^{C}_{EP_{j}}$ derived from the Weibull model, a parametric estimator for the weighted all-cause hazard ratio is given by

8 $$\begin{array}{@{}rcl@{}} \hat\theta^{w}_{CE}(t)=\frac{\sum\limits_{j=1}^{k}{w_{EP_{j}}\cdot \hat\lambda^{I}_{EP_{j}}(t)}}{\sum\limits_{j=1}^{k}{w_{EP_{j}}\cdot \hat\lambda^{C}_{EP_{j}}(t)}}. \end{array} $$

The pre-specification of a survival model to identify the cause-specific hazard must be seen as a considerable restriction as the shape of the survival distribution is usually not known in advance. Thus, it is of interest to evaluate how sensible the parametric estimator reacts when the survival model is miss-specified. Moreover, there is the general interest in deriving a less restrictive non-parametric estimator.

A related variance estimator for (8) cannot easily be deduced and thus an asymptotic distribution of the parametric estimator given in (8) is not available. Therefore, Rauch et al. [15] considered a permutation test to test the null hypothesis specified above. For the permutation test the sampling distribution is built by resampling the observed data. Thereby, the originally assigned treatment groups are randomly assigned to the observation without replacement in several runs. Although this is an elegant option without the need to make further restrictive assumptions, the disadvantage is that such a permutation test is not available as a standard application in statistical software but requires implementation. Moreover, depending on the trial sample size and the computer capacities, this is a very time consuming approach.

#### New point estimator and closed formula test statistic

To derive the new point estimator, we will assume in the following that the baseline hazards for all individual components and for the composite are equivalent within each group, meaning that

$$\begin{array}{@{}rcl@{}} \lambda_{CE,i}(t)&=&\lambda_{0}(t)exp(\beta_{CE}X_{i})\\ &=&\lambda_{0}(t)\sum_{j=1}^{k} exp(\beta_{EP_{j}}X_{i}) \end{array} $$

*i*=1,...,*n*, and thus (4) reads as

$$\begin{array}{@{}rcl@{}} \lambda^{w}_{CE,i}(t)=\lambda_{0}(t)\sum_{j=1}^{k} w_{EP_{j}}exp(\beta_{EP_{j}}X_{i}). \end{array} $$

This is a very restrictive assumption usually not met in practice. The assumption is only required to formally derive the new non-parametric estimator. We do not generally focus on data situations were this assumption is fulfilled. The estimator is only relevant for practical use if deviations from this assumptions produce no relevant bias. This will be investigated in detail in the sections *Simulation scenarios* and *Results*.

Under this assumption, the baseline hazards in the representation of the weighted all-cause hazard ratio cancel out. By this, the weighted all-cause hazard ratio is no longer time-dependent. It is therefore possible to replace the cause-specific hazards by the cumulative cause-specific hazards:

9 $$\begin{array}{@{}rcl@{}} \theta^{w}_{CE}=\theta^{w}_{CE}(t)&=&\frac{\sum\limits_{j=1}^{k} w_{EP_{j}}\lambda^{I}_{EP_{j}}(t)}{\sum\limits_{j=1}^{k} w_{EP_{j}}\lambda^{C}_{EP_{j}}(t)}\notag\\ &=&\frac{\sum\limits_{j=1}^{k} w_{EP_{j}}\lambda_{0}(t)exp(\beta_{EP_{j}}\cdot 1)}{\sum\limits_{j=1}^{k} w_{EP_{j}}\lambda_{0}(t)exp(\beta_{EP_{j}}\cdot 0)}\notag\\ &=&\frac{\sum\limits_{j=1}^{k} w_{EP_{j}}\int_{0}^{t}\lambda_{0}(s)exp(\beta_{EP_{j}})\mathrm{d}s}{\sum\limits_{j=1}^{k} w_{EP_{j}}\int_{0}^{t}\lambda_{0}(s)exp(0)\mathrm{d}s} \notag\\ &=&\frac{\sum\limits_{j=1}^{k} w_{EP_{j}}\Lambda^{I}_{EP_{j}}(t)}{\sum\limits_{j=1}^{k} w_{EP_{j}}\Lambda^{C}_{EP_{j}}(t)}, \end{array} $$

where $\Lambda _{EP_{j}}(t),\ j=1,...,k$, refer to the corresponding cause-specific cumulative hazards over the period [0,*t*]. With *X*_*i*_ equal to 1 if the individual *i* belongs to the intervention and 0 otherwise. This representation can be used to derive a non-parametric estimator for the weighted all-cause hazard ratio using the corresponding non-parametric Nelson-Aalen estimators given as

$$\begin{array}{@{}rcl@{}} \hat\Lambda^{I}_{EP_{j}}(t):=\sum_{t_{l}\leq t}\frac{d_{EP_{j},l}^{I}}{n_{l}^{I}},\quad \hat\Lambda^{C}_{EP_{j}}(t):=\sum_{t_{l}\leq t}\frac{d_{EP_{j},l}^{C}}{n_{l}^{C}}, \end{array} $$

using the notations given in section *Point estimator and test statistic*. By this a non-parametric estimator for the weighted all-cause hazard ratio is given by

10 $$\begin{array}{@{}rcl@{}} \widetilde\theta^{w}_{CE}(t):= \frac{\sum\limits_{j=1}^{k} w_{EP_{j}}\cdot\hat\Lambda^{I}_{EP_{j}}(t)}{\sum\limits_{j=1}^{k}w_{EP_{j}}\cdot\hat\Lambda^{C}_{EP_{j}}(t)}. \end{array} $$

In contrast to the parametric estimator $\hat \theta ^{w}_{CE}(t)$ given in (8), the non-parametric estimator $\widetilde {\theta }^{w}_{CE}(t)$ given in (10) does not require the pre-specification of a survival model. However, the correctness of the non-parametric estimator is still based on the assumption of equal cause-specific baseline hazards. In case the baseline hazards differ, $\widetilde {\theta }^{w}_{CE}(t)$ can be calculated but represents a biased estimator for $\theta ^{w}_{CE}(t)$. Therefore, it is of interest to evaluate how sensible the non-parametric estimator reacts when the equal baseline hazards assumption is violated.

An alternative testing procedure to the discussed permutation test can be formulated by a weight-based log-rank test statistic derived from a modification of the common log-rank test statistic given in (3). We use the expression ‘weight-based log-rank test’ instead of ‘weighted log-rank test’, as in the literature the weighted log-rank test refers to weights which are assigned to the different observation time points whereas we aim to weight the different event types of a composite endpoint.

Under the null hypothesis (7) and under the assumption that the weighted all-cause hazards are equal between groups, the random variable $D_{EP_{j},l}^{I},\ j=1,...,k$, for randomly sampling $d_{EP_{j},l}^{I}$ events of type *EP*_*j*_ from $n_{l}^{I}$ patients where $n_{l}^{I}$ is a subset of the pooled sample with $n_{l}^{I}+n_{l}^{C}$ individuals including a total of *d*_*l*_ events at a fixed time point *t*_*l*_ is hypergeometrically distributed as

$$\begin{array}{@{}rcl@{}} D^{I}_{EP_{j},l}\sim Hyp\left(n_{l}^{I}+n_{l}^{C},d_{EP_{j},l}, d_{EP_{j},l}^{I}\right). \end{array} $$

The expectation of the additive weighted $D^{I}_{EP_{j},l}$ over all distinct *t*_*l*_≤*τ* is given as

$$\begin{array}{*{20}l} &\mathbb{E}\left(\sum_{t_{l}\leq \tau}\sum_{j=1}^{k}w_{EP_{j}}\cdot D_{EP_{j},l}^{I}\right)\\&=\sum_{t_{l}\leq \tau}\sum_{j=1}^{k}w_{EP_{j}}\mathbb{E}\left(D_{EP_{j},l}^{I}\right)\\ &=\sum_{t_{l}\leq \tau}\sum_{j=1}^{k}w_{EP_{j}}\frac{n_{l}^{I}}{n_{l}^{I}+n_{l}^{C}}d_{EP_{j},l}\\&=\sum_{t_{l}\leq \tau}\frac{n_{l}^{I}}{n_{l}^{I}+n_{l}^{C}}\sum_{j=1}^{k}w_{EP_{j}}d_{EP_{j},l} \end{array} $$

and the variance is given as

$$\begin{array}{*{20}l} &Var\left(\sum_{t_{l}\leq \tau}\sum_{j=1}^{k}w_{EP_{j}}\cdot D_{EP_{j},l}^{I}\right)\\ &=\sum_{t_{l}\leq \tau}\sum_{j=1}^{k}w_{EP_{j}}^{2} Var\left(D_{EP_{j},l}^{I}\right)\\ &=\sum_{t_{l}\leq \tau}\sum_{j=1}^{k}w_{EP_{j}}^{2}{\frac{n_{l}^{I} n_{l}^{C} (n_{l}^{I}+n_{l}^{C}- d_{EP_{j},l}) d_{EP_{j},l}}{(n_{l}^{I}+n_{l}^{C})^{2}(n_{l}^{I}+n_{l}^{C}-1)}}\\ &=\sum_{t_{l}\leq \tau}\frac{n_{l}^{I} n_{l}^{C} }{(n_{l}^{I}+n_{l}^{C})^{2}(n_{l}^{I}+n_{l}^{C}-1)} \cdot \\ &\left((n_{l}^{I}+n_{l}^{C})\sum_{j=1}^{k}w_{EP_{j}}^{2}\cdot d_{EP_{j},l} - \sum_{j=1}^{k}w_{EP_{j}}^{2}\cdot d^{2}_{EP_{j},l}\right), \end{array} $$

assuming that no events of different types occur at the same time point.

Thus, the weight-based log-rank test for the proposed weighted effect measure can be defined analogous to (3) as

11 $$\begin{array}{@{}rcl@{}} LR^{w}:=&\\ &\frac{\sum\limits_{t_{i}\leq \tau}{\left(\sum\limits_{j=1}^{k}{w_{EP_{j}} d_{EP_{j},i}^{I}}-\frac{n_{i}^{I}}{n_{i}^{I}+n_{i}^{C}}\sum\limits_{j=1}^{k}{w_{EP_{j}}d_{EP_{j},i}}\right)}} {\sqrt{\sum\limits_{t_{i}\leq \tau}\left(\frac{{n_{i}^{I} n_{i}^{C}\left(\left(n_{i}^{I}+n_{i}^{C}\right)\sum\limits_{j=1}^{k}{w_{EP_{j}}^{2}d_{EP_{j},i}}-\sum\limits_{j=1}^{k}{w_{EP_{j}}^{2} d^{2}_{EP_{j},i}}\right)}}{\left(n_{i}^{I}+n_{i}^{C}\right)^{2}\left(n_{i}^{I}+n_{i}^{C}-1\right)}\right)}}. \end{array} $$

Under the null hypothesis of equal weighted composite (cumulative) hazards the test statistic (11) is approximately standard normal distributed. Hence, the null hypothesis is rejected if *LR*^*w*^≤−*z*_1−*α*_, where *z*_1−*α*_ is the corresponding (1−*α*)-quantile of the standard normal distribution and *α* is the one-sided significance level.

Note that the common weighted log-rank test can be shown to be equivalent to the Cox score test [16] because the weights are working on the coefficient *β* and thus the partial likelihood and its logarithm can be easily deduced. The intention of the common weighted log-rank test is to weight the time points. However, in our weight-based log-rank test, the weights have another meaning and are working on the whole hazard not only on the coefficient. Thus, the log-likelihood translates to a form were the weights are additive and therefore the score test does not translate to the test statistic proposed in this work. This was also the reason why we called our test ’weight-based’ and not ’weighted’ log-rank test. Our test is valid but must be interpreted as a Wald-type test statistic.

#### Step-by-step guidance for the choice of weights

When using the weighted all-cause hazard ratio as the efficacy effect measure for a composite endpoint it is important to fix the weights in the planning stage of the study. This can be seen as a quite challenging task, as the choice of the weights importantly influences the final outcome and the interpretation of the results. Thus, it is important to choose the weights in a well-reflected way and not arbitrarily. To help researchers with this task, we provide detailed steps on how to choose appropriate weights for a specific clinical trial situation. When discussing the choice of weights, it must be kept in mind that by using the standard all-cause hazard, i.e. the unweighted scenario, this corresponds to equal weights for all components implying that event types with a higher event frequency are naturally up-weighted. Therefore, equal weights for all components can be considered at least as arbitrary as predefined weights according to relevance considerations. To define reasonable weights, we first recall the weighted all-cause hazard function as introduced in (4)

$$\begin{array}{@{}rcl@{}} \lambda^{w}_{CE}(t)=\sum_{j=1}^{k}w_{EP_{j}}\cdot \lambda_{EP_{j}}(t), \ j=1,..,k. \end{array} $$

The weighted all-cause hazard can also be interpreted as the standard all-cause hazard based on modified cause-specific hazards $\tilde {\lambda }_{EP_{j}}(t)$ where

$$\begin{array}{@{}rcl@{}} \tilde{\lambda}_{EP_{j}}(t):=w_{EP_{j}}\cdot \lambda_{EP_{j}}(t), \ j=1,..,k. \end{array} $$

Thus, by introducing the component weights we implicitly modify the event time distribution that is the corresponding survival function. When choosing a weight unequal to 1, the survival distribution changes its shape. For a weight larger than 1, the number of events artificially increases and as a consequence, the survival function decreases sooner. In contrast, for a weight smaller than 1 the survival distribution becomes more flat as the number of events is artificially decreased. Whereas the all-cause hazard ratio can be heavily masked by a large cause-specific hazard of a less relevant component, a more relevant component with a lower number of events can only have a meaningful influence on the composite effect measure, when it is up-weighted (or if the less relevant component is down-weighted accordingly). On the contrary, if a large cause-specific hazard is down-weighted this can result in a power loss. Therefore, weighting can improve interpretation but the effect on power can be positive or negative, depending on the data situation at hand.

In order to preserve the comparability to the unweighted all-cause hazard ratio, we recommend to fix the weight of the most important component, which is often given by ‘death’, to 1. All other weights should then be chosen smaller or equal to 1. When considering a weight for the most relevant component larger than 1, this results in endless possibilities and it becomes more difficult to set the weights for the other less relevant events in an adequate relation. The general recommendation of fixing all weights $w_{EP_{j}}\leq 1,\ j=1,...,k$ is moreover reasonable because choosing a set of weights which are both - smaller and greater than 1 - can cause a situation where the weighted all-cause hazard is equivalent to the standard all-cause hazard. This is problematic because in this case we cannot differentiate if the effect is due to the weighting scheme or due to the underlying cause-specific hazards. For illustration of the latter problem, consider two event types *EP*_1_ and *EP*_2_ with exponentially distributed event times, where *EP*_1_ corresponds to the more relevant endpoint

$$\begin{array}{@{}rcl@{}} \lambda_{EP_{1}}(t)=0.2 \qquad \lambda_{EP_{2}}(t)=0.3. \end{array} $$

This leads to the standard all-cause hazard

$$\begin{array}{@{}rcl@{}} \lambda_{CE}(t)=0.2+0.3=0.5. \end{array} $$

If the weights are chosen as $w_{EP_{1}}=1.3$ and $w_{EP_{2}}=0.8$ the weighted cause-specific hazards are given as

$$\begin{array}{*{20}l} &\tilde\lambda_{EP_{1}}(t)=1.3\cdot 0.2=0.26 \\ &\tilde\lambda_{EP_{2}}(t)=0.8\cdot 0.3=0.24 \end{array} $$

and therefore, the weighted all-cause hazard is equivalently given by

$$\begin{array}{@{}rcl@{}} \lambda^{w}_{CE}(t)=0.26+0.24=0.5. \end{array} $$

Choosing the weights $w_{EP_{1}}=1$ and $w_{EP_{2}}=0.6$ gives the weighted cause-specific hazards

$$\begin{array}{*{20}l} &\tilde\lambda_{EP_{1}}(t)=1\cdot 0.2=0.2 \\ &\tilde\lambda_{EP_{2}}(t)=0.6\cdot 0.3=0.18, \end{array} $$

and therefore

$$\begin{array}{@{}rcl@{}} \lambda_{CE}^{w}(t)=0.2+0.18=0.38, \end{array} $$

where the influence of the weights is now visible. Instead of interpreting the weighted hazards, for the applied researcher it might be easier to consider the corresponding weighted composite survival function $S_{CE}^{w}(t)$ given as

$$\begin{array}{@{}rcl@{}} S_{CE}^{w}(t)&=&exp(-\Lambda_{CE}^{w}(t))=exp\left(-\int_{0}^{t}\lambda_{CE}^{w}(x)dx\right)\\ &=&exp\left(-\int_{0}^{t} \left(\sum_{j=1}^{k}w_{EP_{j}}\cdot \lambda_{EP_{j}}(x)\right) dx\right)\\&=&exp\left(- \sum_{j=1}^{k} w_{EP_{j}}\int_{0}^{t} \left(\lambda_{EP_{j}}(x)\right) dx\right)\\ &=&exp\left(- \sum_{j=1}^{k} w_{EP_{j}}\Lambda_{EP_{j}}(t)\right)\\ &=&\prod_{j=1}^{k} exp(-w_{EP_{j}}\Lambda_{EP_{j}}(t)). \end{array} $$

It can be seen that the weights are still working multiplicatively on the cumulative cause-specific hazards and the event time distributions for the different event types are also connected multiplicatively. By the introduction of the weights we still assume that an individual can only experience one event but (for weights smaller than 1) less individuals experience the event. This means that the expected number of events decreases with a weight smaller than 1. Therefore, the weighted survival function for the composite still corresponds to a time to first event setting but with a proportion of events which is lower compared to the unweighted approach.

**Fig. 1:**
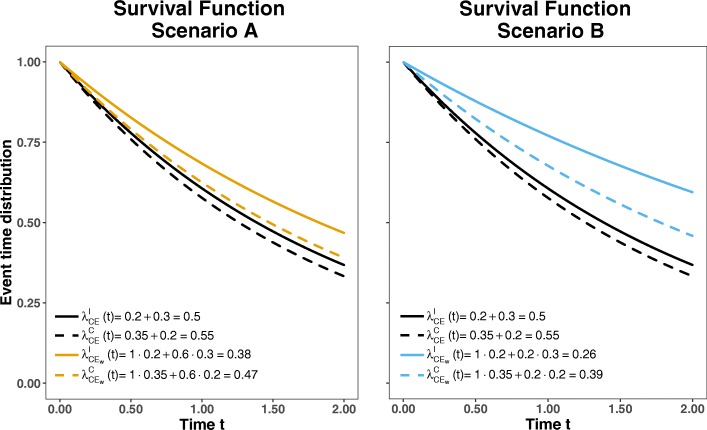
Event time distributions for two different weighting schemes: Scenario A: $w_{EP_{1}}=1, w_{EP_{2}}=0.6$; Scenario B: $w_{EP_{1}}=1, w_{EP_{2}}=0.2$

Comparing the graphs of the weighted and unweighted event time distributions can be a helpful tool for choosing the weights as shown in Fig. 1 for the exemplary setting discussed above. It can be seen that both weighting schemes yield a larger difference between the event time distributions when comparing the intervention versus the control, however the second weighting scheme shows the larger difference.

In conclusion, we recommend to proceed as follows in order to choose the weights

1. Identify the clinically most relevant event type (e.g. ‘death’) and assign a weight of 1.
2. Choose the order of clinical relevance for the remaining event types. For each event type *EP*_*j*_ you should answer the question "How many events of type *EP*_*j*_ can be considered as equally harmful than observing one event (or any other amount of reference events) in the clinically most relevant endpoint?". For example, if in the example given above 5 events of type *EP*_2_ are considered as equally harmful as one event of *EP*_1_, then the weighting scheme proposed in Scenario B might be preferred. If instead the researcher arguments that 5 events of type *EP*_2_ are considered as equally harmful as 3 events of *EP*_1_, then the weighting scheme proposed in Scenario A should be preferred. The weights are thus mend to bring all events to the same severity scale. By assigning a weight of 1 to the most relevant event type, this event type acts as the reference event. Therefore, the weighted survival function and its summarizing measures (median survival, hazard ratio) can be interpreted as a standard survival function for the reference event. For example, if ’death’ is the reference event, on a population and on an individual patient level, the weighted survival function then expresses the probability to be neither dead nor in a condition considered as equally harmful. The median weighted survival can be interpreted as the time when half of the population is either dead or in an equally harmful condition.
3. If there are some assumption about the form of the underlying event time distributions, then the functional form of the cause-specific hazards is known. The weighted cause-specific hazards are obtained by simple multiplication with the weighting factors. We recommend to choose several weighting scenarios and to plot the resulting weighted and unweighted event time distributions and to investigate graphically how different weights would affect the expected survival time and median survival per group. Moreover, the weighted and unweighted hazard ratio can be analytically deduced and compared. By this, the impact of the weighting scheme becomes more explicit.

### Simulation scenarios

To provide a systematic comparison of the original parametric estimator $\widehat \theta ^{w}_{CE}(t)$ to the new non-parametric estimator $\widetilde \theta ^{w}_{CE}(t)$ for the weighted all-cause hazard ratio and in order to analyse the performance of the weight-based log-rank test compared to the originally proposed permutation test we performed a simulation study with the software R Version 3.3.3 [17].

**Fig. 2:**
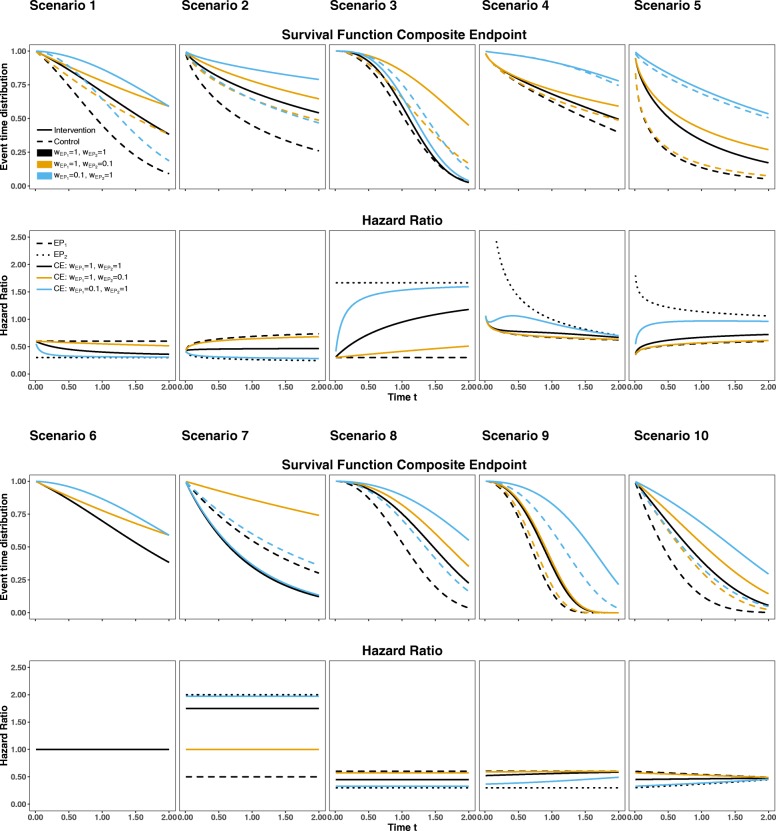
Event time distributions for the intervention (dashed lines) and control (solid lines) for the composite endpoint based on the unweighted (black lines) and weighted (yellow and blue lines) cause-specific hazards as well as the unweighted all-cause hazard ratio (black solid line) in comparison to the weighted all-cause hazard ratios (yellow and blue lines) and the cause-specific hazard ratios (dotted black lines)

Within our simulation study, we investigate various data scenarios for a composite endpoint composed of two components *EP*_1_ and *EP*_2_. We restrict our simulations to weights given by $w_{EP_{1}}=1,\ w_{EP_{2}}=0.1$ or $w_{EP_{1}}=0.1,\ w_{EP_{2}}=1$ where the two event types are thus considered to show a considerable difference in clinical relevance. The results for another less extreme weighting scheme are provided as Additional file 1 (i.e. weights 1 and 0.7). A total of 10 scenarios based on different underlying hazard functions were considered in order to mimic situations where the underlying assumptions of both approaches are fulfilled and those where they are (partly) violated. For the original parametric estimator, the cause-specific hazards were estimated by fitting Weibull models. A total of 1000 data sets each with *n*=200 patients (100 patients per group) were simulated for each scenario. The amount of simulated data sets was limited to 1000 because of the time-consuming runtime of the permutation test which was based on 1000 runs. We used the pseudo-random generator Mersenne Twister [18]. For simulating the underlying event times, the approach described by Bender et al. [19] was used. The minimal follow-up was either fixed to *τ*=1 or *τ*=2 year(s). For each scenario the methods were compared on the same data sets. In case of non-convergence of a model, the data set was excluded. Table 1 lists the underlying hazard functions for the different simulation scenarios and summarizes briefly which assumptions are met. In Fig. 2 the corresponding weighted and unweighted event time distributions for the composite for the intervention and the control group are graphically displayed for all 10 scenarios. In addition, the related weighted and unweighted hazard ratios for the composite are visualized along with unweighted cause-specific effects.

**Table 1:** Investigated simulation scenarios

Scenario	$\lambda ^{I}_{EP_{1}}(t)$	$\lambda ^{C}_{EP_{1}}(t)$	$\lambda ^{I}_{EP_{2}}(t)$	${\lambda ^{C}_{EP_{2}}(t)}$	Description	Assumptions for original parametric estimator ^∗^	Assumptions for new non-parametric estimator ^*#*^
1	0.24	0.4	0.24*t*	0.8*t*	Weibull distributed;	$\checkmark $	✗
					PH assumption only fulfilled for components;		
					unequal cause-specific baseline hazards		
2	0.192*t*^−0.2^	0.28*t*^−0.3^	0.084*t*^−0.3^	0.32*t*^−0.2^	Weibull distributed;	✗	✗
					PH assumption not fulfilled for components and composite;		
					unequal cause-specific baseline hazards		
3	0.24*t*	0.8*t*	1.2*t*^2^	0.72*t*^2^	Weibull distributed;	$\checkmark $	✗
					PH assumption only fulfilled for components;		
					unequal cause-specific baseline hazards		
4	0.2*t*^−0.4^	0.3*t*^−0.3^	0.1*t*	0.1*t*^1.5^	Weibull distributed;	✗	✗
					PH assumption not fulfilled for components and composite;		
					unequal cause-specific baseline hazards		
5	0.5*t*^−0.4^	0.9*t*^−0.5^	0.25	0.22*t*^0.1^	Weibull distributed;	✗	✗
					PH assumption not fulfilled for components and composite;		
					unequal cause-specific baseline hazards		
6	0.24	0.24	0.24*t*	0.24*t*	Weibull distributed;	$\checkmark $	✗
					PH assumption fulfilled for components and composite;		
					unequal cause-specific baseline hazards		
7	0.05	0.1	1	0.5	Weibull distributed;	$\checkmark $	$\checkmark $
					PH assumption fulfilled for components and composite;		
					equal cause-specific baseline hazards		
8	0.42*e*^0.7*t*^−0.42	0.7*e*^0.7*t*^−0.7	0.21*e*^0.7*t*^−0.21	0.7*e*^0.7*t*^−0.7	Gompertz distributed;	✗	$\checkmark $
					PH assumption fulfilled for components and composite;		
					equal cause-specific baseline hazards		
9	0.42*e*^2*t*^−0.42	0.7*e*^2*t*^−0.7	0.21*e*^0.7*t*^−0.21	0.7*e*^0.7*t*^−0.7	Gompertz distributed;	✗	✗
					PH assumption only fulfilled for components;		
					unequal cause-specific baseline hazards		
10	0.42*e*^0.7*t*^	0.7*e*^0.8*t*^	0.21*e*^0.8*t*^	0.7*e*^0.6*t*^	Gompertz distributed;	✗	✗
					PH assumption not fulfilled for components and composite;		
					unequal cause-specific baseline hazards		

$\lambda ^{I}_{EP_{1}}(t),\lambda ^{C}_{EP_{1}}(t),\lambda ^{I}_{EP_{2}}(t),\lambda ^{C}_{EP_{2}}(t)$: cause-specific hazard functions for *EP*_1_ and *EP*_2_ in the intervention and the control group, respectively; PH: proportional hazards;

^*^It is assumed that the Weibull model used to estimate the cause-specific hazards is the correct one and the PH assumption is fulfilled for the components;

^#^It is assumed that the cause-specific baseline hazards are equal.

For Scenario 1-7 the cause-specific hazards are Weibull, or exponentially, distributed with the hazard of the form [20]

12 $$ \lambda(t)=\kappa\cdot \nu\cdot t^{\nu-1}.  $$

Thereby, *κ*>0 is the scale parameter and *ν*>0 is the shape parameter. The investigated scenarios show to some extend the flexibility of the Weibull model. Situations with earlier occurring events for one event type (higher cause-specific hazard) and later occurring events for the other event type (lower cause-specific hazards) are capture as well as situations where the difference in hazards is smaller. In the scenarios 1-6 at least one cause-specific hazard is time-dependent whereas in Scenario 7 the hazards are constant.

The hazard for the composite increases over time for the Scenarios 1 and 3 and decreases for Scenario 2. For the Scenarios 4 and 5 the hazard first decreases and then increases after a while. For the Scenarios 1 and 3 the proportional hazards assumption is fulfilled for each of the event types simultaneously. Also note that in Scenario 3 and partly in the Scenarios 4 and 5 the effects for the event types point into opposite directions. Scenario 6 depicts a situation where no treatment effect for the individual components and the composite exists. In Scenario 7 there are opposite effects for the individual components which cancel out in the combined composite for one weighting scheme.

As we aim to quantify how robust the original parametric estimator for the weighted all-cause hazard ratio based on the Weibull model is when the event times for the components in fact *do not* follow a Weibull distribution, Scenarios 8 to 10 are based on a Gompertz distribution. Like the Weibull model the Gompertz model fulfils the proportional hazards assumptions where the hazard is parametrized as

13 $$ \lambda(t)=\kappa\cdot e^{\nu\cdot t}+\epsilon,   $$

which is also referred to the Gompertz-Makeham distributed hazard [20, 21]. Again, *κ*>0 is a scaling parameter and *ν*>0 a shape parameter. In addition, a more general term *ε*≥−*κ* defining the intercept is formulated. For all Scenarios with Gompertz distributed event times the hazard for the composite increases over time. For the situation where the shape parameters are equal across all event types the proportional hazards assumption does apply to the composite. This is the case for Scenario 8 but not for the Scenarios 9 and 10. The proportional hazards assumption also holds true for each event type separately for the Scenarios 8 and 9. In Scenario 10 the proportional hazards assumption is violated for all event types and for the composite.

## Results

Table 2 displays the results of the simulation study for all Scenarios 1 to 10. Columns 2 and 3 present check boxes for the underlying model assumptions of the two estimators. Especially for those scenarios where some assumptions are violated, we are interested in the (standardized) bias, the relative efficiency, and the coverage of the corresponding confidence interval for the different estimators (see Table 3). Thereby. the bias is quantified by comparing the mean logarthmized (natural) estimators (Table 2 Columns 10 and 11) to the corresponding natural logarithm of the true effect (Table 2 Column 7) which is fixed by the simulation setting. For the standardized bias the bias is divided by the corresponding standard error of the estimated effects. The relative efficiency is the quotient of the mean square error of the original estimator divided by the mean square error of the non-parametric estimator. A relative efficiency smaller than one is in favour of the original estimator. The mean square error is the sum of the quadratic bias and the quadratic standard error for the logarithmized estimators. The coverage is the proportion of times the 95% confidence interval for each estimated effect includes the true effect. To determine the confidence intervals, the standard error for all estimated effects is required which we obtained by the permutation distribution. Thereby, again logarithmic scale is used so that the estimators’ distribution is not skewed and thus their standard deviation and the performance measures in Table 3 can be interpreted. In Column 4 of Table 2 the time point *τ* at which the estimators are evaluated is shown and Columns 5 and 6 show the underlying component weights. Note that by switching the weights between the two components, we implicitly investigate the influence of all hazard combinations when the relevance of the components is reversed. Column 7 displays the logarithmized true weighted all-cause hazard ratio at time *τ* which can be obtained from the underlying cause-specific hazard functions. Columns 8 and 9 show the mean amount of events averaged over all data sets per scenario for all event types separately and its standard deviation. Columns 10 and 11 show the mean of the logarithmized estimated weighted all-cause hazard and its standard deviation based on the original parametric estimator $\widehat {\theta }^{w}_{CE}(\tau)$ and based on the new non-parametric estimator $\tilde {\theta }^{w}_{CE}(\tau)$. Columns 12 to 13 show the empirical power values for the originally proposed permutation test based on $\widehat {\theta }^{w}_{CE}(\tau)$ and for the new weight-based log-rank test based on $\tilde {\theta }^{w}_{CE}(\tau)$. Note that the reported power values correspond to one-sided tests based on a one-sided significance level of 0.025. Table 3 depicts the amount of simulations that converged (Columns 2 and 3), the bias (Columns 4 and 5), the standardized bias (Columns 6 and 7), the square root of the mean square error (Columns 8 and 9), the relative efficiency (Column 10), and the coverage (Columns 11 and 12) for the logarithmized original and new estimators.

**Table 2:** Simulation results

Sc.	Assumptions for	Assumptions for	**τ**	Weights		Ln of	Mean number		Mean of estimated		Power for	
	original parametric	new non-parametric				True	of events (sd)		Ln(WHR) (sd)		permutation	weight-based
	estimator^*^	estimator^#^				WHR					test	log-rank test
				$w_{EP_{1}}$	$w_{EP_{2}}$	$ln(\theta _{CE}^{w}(\tau))$	*E* *P*_1_	*E* *P*_2_	$ln(\hat {\theta }^{w}_{CE}(\tau))$	$ln(\tilde {\theta }^{w}_{CE}(\tau))$	$\hat {\theta }^{w}_{CE}(\tau)$	$\tilde {\theta }^{w}_{CE}(\tau)$
1a	$\checkmark $	✗	1	1	0.1	-0.60	50.21 (6.51)	35.16 (5.21)	-0.61 (0.27)	-0.57 (0.28)	0.66	0.72
1b			2	1	0.1	-0.67	72.60 (6.74)	80.13 (6.73)	-0.67 (0.20)	-0.60 (0.24)	0.94	0.90
1c			2	0.1	1	-1.18			-1.20 (0.22)	-1.14 (0.21)	1.00	1.00
2a	✗	✗	1	1	0.1	-0.44	48.22 (6.08)	37.15 (5.33)	-0.59 (0.29)	-0.58 (0.29)	0.58	0.75
2b			2	1	0.1	-0.38	67.79 (6.75)	51.93 (5.60)	-0.54 (0.23)	-0.53 (0.23)	0.64	0.81
3a	$\checkmark $	✗	1	1	0.1	-0.88	39.97 (5.63)	47.94 (5.87)	-0.90 (0.29)	-1.00 (0.31)	0.90	0.96
3b			1	0.1	1	0.43			0.42 (0.29)	0.38 (0.28)	0.00	0.00
3c			2	1	0.1	-0.68	69.84 (6.39)	124.63 (6.40)	-0.67 (0.20)	-0.80 (0.21)	0.96	1.00
4a	✗	✗	1	1	0.1	-0.39	62.84 (6.39)	6.49 (2.50)	-0.22 (0.64)	-0.23 (0.26)	0.14	0.29
4b			1	0.1	1	-0.08			0.12 (0.96)	0.01 (0.44)	0.02	0.05
4c			2	1	0.1	-0.46	86.66 (6.95)	24.29 (4.57)	-0.27 (0.21)	-0.28 (0.21)	0.27	0.44
4d			2	0.1	1	-0.36			-0.12 (0.40)	-0.15 (0.32)	0.05	0.17
5a	✗	✗	1	1	0.1	-0.56	133.27 (6.26)	19.31 (4.05)	-0.82 (0.28)	-0.82 (0.17)	1.00	1.00
5b			1	0.1	1	-0.03			-0.04 (0.57)	-0.14 (0.30)	0.03	0.34
6a	$\checkmark $	✗	2	1	0.1	0.00	67.04 (6.74)	56.49 (6.37)	0.00 (0.21)	0.00 (0.23)	0.02	0.08
6b			2	0.1	1	0.00			-0.00 (0.25)	-0.00 (0.24)	0.02	0.06
7a	$\checkmark $	$\checkmark $	2	1	0.1	0.00	15.83 (3.84)	141.89 (6.18)	0.01 (0.30)	0.02 (0.28)	0.02	0.02
7b			2	0.1	1	0.68			0.68 (0.16)	0.68 (0.17)	0.00	0.00
8a	✗	$\checkmark $	2	1	0.1	-0.56	108.25 (6.93)	78.79 (6.69)	-0.69 (0.21)	-0.56 (0.19)	0.92	0.96
8b			2	0.1	1	-1.12			-1.29 (0.24)	-1.13 (0.21)	1.00	1.00
9a	✗	✗	1	0.1	1	-0.88	132.78 (6.43)	15.22 (3.56)	-1.10 (0.42)	-0.93 (0.31)	0.77	0.97
9b			2	1	0.1	-0.51	178.24 (4.21)	21.76 (4.21)	-0.66 (0.18)	-0.52 (0.15)	0.96	0.97
9c			2	0.1	1	-0.71			-1.31 (0.48)	-0.89 (0.30)	0.90	0.99
10a	✗	✗	1	1	0.1	-0.64	84.95 (7.02)	62.44 (6.34)	-0.58 (0.21)	-0.59 (0.21)	0.81	0.94
10b			1	0.1	1	-0.95			-1.04 (0.23)	-1.05 (0.23)	1.00	1.00
10c			2	1	0.1	-0.72	113.64 (6.92)	80.58 (6.55)	-0.55 (0.17)	-0.61 (0.18)	0.89	0.98
10d			2	0.1	1	-0.79			-0.95 (0.19)	-1.03 (0.21)	1.00	1.00

Ln: natural logarithm; WHR: Weighted all-cause hazard ratio; sd: Standard deviation;

^*^It is assumed that the Weibull model used to estimate the cause-specific hazards is the correct one;

^#^It is assumed that the cause-specific baseline hazards are equal.

**Table 3:** Simulation results: Performance

Sc.	Amount of Simulations		Bias		Standardized Bias		$\sqrt {\text {Mean Square Error}}$		Relative Efficiency	Coverage ^∗^	
	$ln(\hat {\theta }^{w}_{CE}(\tau))$	$ln(\tilde {\theta }^{w}_{CE}(\tau))$	$ln(\hat {\theta }^{w}_{CE}(\tau))$	$ln(\tilde {\theta }^{w}_{CE}(\tau))$	$ln(\hat {\theta }^{w}_{CE}(\tau))$	$ln(\tilde {\theta }^{w}_{CE}(\tau))$	$ln(\hat {\theta }^{w}_{CE}(\tau))$	$ln(\tilde {\theta }^{w}_{CE}(\tau))$	$\frac {MSE\left (ln(\hat {\theta }^{w}_{CE}(\tau))\right)}{MSE\left (ln(\tilde {\theta }^{w}_{CE}(\tau))\right)}$	$ln(\hat {\theta }^{w}_{CE}(\tau))$	$ln(\tilde {\theta }^{w}_{CE}(\tau))$
1a	991	1000	-0.01	0.03	-0.05	0.11	0.27	0.29	0.91	93.14	92.70
1b	997	1000	-0.01	0.07	-0.04	0.30	0.20	0.23	0.76	94.68	93.10
1c	997	1000	-0.02	0.04	-0.07	0.19	0.23	0.22	1.12	95.29	94.80
2a	1000	1000	-0.15	-0.14	-0.51	-0.49	0.32	0.32	1.02	90.60	91.00
2b	999	1000	-0.15	-0.15	-0.63	-0.63	0.28	0.28	1.02	89.09	88.80
3a	1000	1000	-0.02	-0.12	-0.07	-0.37	0.30	0.33	0.79	93.50	91.40
3b	1000	1000	-0.00	-0.04	-0.01	-0.15	0.30	0.29	1.06	94.20	95.20
3c	1000	1000	0.00	-0.12	0.02	-0.58	0.20	0.24	0.68	93.10	88.70
4a	993	1000	0.17	0.16	0.26	0.39	0.66	0.31	4.57	90.07	89.53
4b	993	1000	0.20	0.09	0.21	0.21	0.98	0.46	4.85	94.44	95.47
4c	996	1000	0.19	0.18	0.93	0.84	0.29	0.28	1.03	82.90	84.87
4d	996	1000	0.24	0.21	0.60	0.65	0.46	0.39	1.43	90.14	89.48
5a	995	1000	-0.26	-0.25	-0.91	-1.46	0.38	0.31	1.55	71.80	68.07
5b	995	1000	-0.01	-0.11	-0.01	-0.35	0.57	0.32	3.21	93.56	93.99
6a	998	998	0.00	0.00	0.01	0.01	0.21	0.23	0.86	94.79	94.60
6b	998	998	-0.00	-0.00	-0.01	-0.01	0.25	0.24	1.11	95.69	95.90
7a	984	1000	0.01	0.02	0.04	0.07	0.30	0.28	1.16	94.60	95.20
7b	984	1000	0.01	0.01	0.04	0.03	0.16	0.17	0.99	94.70	94.99
8a	1000	1000	-0.14	0.00	-0.64	0.01	0.25	0.19	1.78	89.20	93.70
8b	1000	1000	-0.17	-0.01	-0.70	-0.05	0.30	0.21	1.97	85.00	93.30
9a	998	998	-0.22	-0.05	-0.53	-0.18	0.47	0.32	2.22	90.68	96.09
9b	990	1000	-0.15	-0.01	-0.81	-0.04	0.23	0.15	2.29	86.97	94.20
9c	990	1000	-0.60	-0.18	-1.25	-0.59	0.77	0.35	4.74	62.93	87.10
10a	1000	1000	0.06	0.04	0.28	0.21	0.21	0.21	1.00	93.70	93.80
10b	1000	1000	-0.09	-0.10	-0.38	-0.44	0.24	0.25	0.94	92.70	92.10
10c	1000	1000	0.17	0.11	0.99	0.60	0.24	0.22	1.27	83.80	89.30
10d	1000	1000	-0.16	-0.23	-0.81	-1.10	0.25	0.32	0.63	87.20	75.90

^*^Defined as proportion of times the 95%-confidence interval for the estimator includes the true effect.

Scenarios 1 and 3 reflect situations where the proportional hazards assumption is fulfilled for each component but the Weibull distributed cause-specific hazards are unequal and thus the composite effect is time-dependent. Since in this scenarios the assumptions for the original estimator is fulfilled it is intuitive that the (standardized) bias is small for the parametric estimator. Although the assumptions for the non-parametric estimator are violated the bias is still rather small. This good performance is also captured in the coverage which is mostly near the anticipated 95%. It is furthermore intuitive that the original estimator shows most often a smaller mean square error in relation to the non-parametric estimator. Note that in Scenario 3 the unweighted effects point into different directions but the direction of the weighted effect depends on the weighting scheme. In the Scenarios 2, 4, and 5 the proportional hazards assumption is not fulfilled for neither the components nor for the composite but the cause-specific hazards still follow a Weibull distribution. For Scenario 2 it can be seen that the estimated weighted effects are the same for both estimators but do not approach the true effect as good as in the Scenarios 1 and 3. This is because both approaches need at least the assumption of proportional hazards in the components. A similar outcome would be expected for the Scenarios 4 and 5. However, in both scenarios the parametric estimator performs much worse than the non-parametric estimator. This is due to the higher variability in the estimations. For Scenario 6 where there is no effect for the unweighted composite both approaches perform quite well. For the original estimator this was expected since its assumptions are fulfilled. In Scenario 7 with the weights 1 for event type 1 and 0.1 for event type 2 the true combined treatment effect is 0. This is also captured quite well in both estimators. Note that only for this specific weighting scheme the composite effect is 0 but not for the other weighting schemes. However, the performance of the estimation approaches is also satisfying for the other weighting schemes. In Scenario 8, Gompertz-Makeham distributed cause-specific hazards are assumed. Thereby, the proportional hazards assumption is fulfilled for the components and the composite. Thus, it is intuitive that the new non-parametric estimator closely coincide with the true effect. However, the parametric estimator based on the Weibull model is relevantly biased independent of the weighting scheme and shows a higher variability. Scenario 9 still depicts Gompertz-Makeham distributed cause-specific hazards but the proportional hazards assumption is only fulfilled for the components and not for the composite. Although the cause-specific baseline hazards are thus unequal the non-parametric estimator performs better in this scenario whereas the parametric estimator shows substantial bias and variability which might be also due to convergence problems. Scenario 10 represents Gompertz distributed cause-specific hazards where the proportional hazards assumption is neither fulfilled for the components nor for the composite. Compared to the two previous scenarios the performance of the parametric estimator has increased and is not globally worse than that of the non-parametric estimator. The performance depends on the weighting scheme. In here, not all *τ*-weight-combinations are displayed. However, the performance of the missing combination scenarios is comparable to the corresponding scenarios displayed.

In conclusion, the original parametric estimator turns out to be sensible against model miss-specifications for estimating the underlying cause-specific hazards as expressed by most values of the (standardized) bias and the coverage of the confidence intervals for Scenarios 4, 5, 8, 9, 10. In these scenarios, the performance of the non-parametric estimator tends to be better because not only the (standardized) bias is smaller and the coverage probability is better but also the relative efficiency favours the non-parametric approach. Moreover, in Scenarios 4 and 5 the (standardized) bias of the parametric estimator is smaller and its variation is considerably higher which cannot only be explained by the smaller amount of converged simulations. The higher amount of non-converging models for the original approach is furthermore a disadvantage. In scenarios where the assumption for the parametric estimator is fulfilled (Scenarios 1 and 3) its performance tends to be better than for the non-parametric approach. Although in these scenarios the assumption of equal cause-specific baseline hazards is violated, the performance of the non-parametric estimator is however not considerably worse than for the parametric estimator.

Except for Scenario 1b, the power of the weight-based log-rank test is uniformly equal or larger than the power of the permutation test. This power advantage in particular occurs in situations where the two point estimators coincide (Scenarios 2a and 2b or 10a) or even when the non-parametric estimator suggests a less extreme effect (Scenarios 8 or 9). For Scenario 6 where there is no effect for the components nor for the composite the permutation test in the investigated scenarios performs better in terms of preserving the type one error. In Scenario 7 where the composite effect is 0 for one weighting scheme the type one error is preserved for the permutation test as well as the weight-based log-rank test in this scenario.

If the weights are chosen to be 1 and 0.7, the performance comparisons basically come to the same results (compare Additional file 1). Summarizing the results of our simulation, the new non-parametric estimator and the corresponding weight-based log-rank test outperform the original estimator and the permutation test.

## Discussion

In this work, we investigated a new estimator and test for the weighted all-cause hazard ratio which was recently proposed by Rauch et al. [15] as an alternative effect measure to the standard all-cause hazard ratio to assess a composite time-to-event endpoint. The weighted all-cause hazard ratio as a weighted effect measure for composite endpoints is appealing because it is a natural extension of the all-cause hazard ratio. It allows to regulate the influence of event types with a greater clinical relevance and thereby eases the interpretation of the results. Generally it must be noted that the weighted all-cause hazard ratio was introduced to ease the interpretation of the effect in terms of clinical relevance. The aim of the weighted effect measure is *not* to decrease the sample size or increase the power. The power of the weighted all-cause hazard ratio can be larger but may also be smaller than the power of the unweighted standard approach.

The original parametric estimator proposed by Rauch et al. [15] requires the specification of a parametric survival model to estimate the cause-specific hazards. Moreover, in the original work by Rauch et al. [15] a permutation test was proposed to test the new effect measure which comes along with a high computational effort. In this work, we overcome these shortcoming by proposing a new non-parametric estimator for the weighted all-cause hazard ratio and a closed formula-based test statistic which is given by a weight-based version of the well-known log-rank test.

The simulation study performed within this work shows that the original parametric estimator is sensible to miss-specifications of the underlying cause-specific event time distribution. If there are uncertainties about the underlying parametric model for the identification of the cause-specific hazards we therefore recommend to use the new non-parametric estimator. In fact, the new non-parametric estimator proposed in this work turns out to be more robust even if the required assumption of equal cause-specific baseline hazards is not met. The relative efficiency as well as the coverage depict also that the performance of the non-parametric estimator is in most cases at least as good as the original parametric estimator. Additionally, in our scenarios convergence problems arose more often when using the parametric estimator. This problems in convergence arose in scenarios where the effect of one event type was either very high at the beginning of the observational period or there was nearly no effect at the end of the observational period where the survival function reaches 0. Moreover, the simulation study shows that the new weight-based log-rank test results in considerably better power properties than the originally proposed permutation test in almost all investigated scenarios. In some scenarios the type one error might not be preserved and it has to be further investigated in which this is exactly the case and how it can be addressed. In addition, the weight-based log-rank test is computationally much less expensive. However, one remaining restriction is that confidence intervals cannot be directly provided because the testing procedure is not equivalent to the Cox score test. The only possibility to provide confidence intervals for the weighted hazard ratio would be by means of bootstrapping techniques.

Apart from investigating the performance of the point estimator and the related statistical test, we additionally provide a step-by-step guidance on how to choose the relevance weights for the individual components in the planning stage. It is often criticized that the choice of relevance weights in a weighted effect measure is to a certain extend arbitrary. By applying our step-by-step guidance for the choice of weights, this criticism can be addressed. To be concrete, we propose to choose a weight of 1 for the clinically most relevant component and to choose weights smaller or equal to 1 for all other components by judging how many events of a certain type would be considered as equally harmful than an event in the most relevant component. Using this approach for defining the weights, comparability to the unweighted approach is given and the most relevant event serves as a reference. When the shape of the different event time distributions is known in the planning stage, we also recommend to look at the plots of the weighted and unweighted event time distributions for different weight constellations to visually inspect the influence of the weight choice on the shape of the survival curves and on the treatment effect.

## Conclusion

In conclusion, we recommend to use the new non-parametric estimator along with the weight-based log-rank test to assess the weighted all-cause hazard ratio. When applying the weighting scheme proposed within our step-by-step guidance, the choice of the weights can be motivated with reasonable clinical knowledge. With the results from this work, the weighted average hazard ratio therefore becomes a very attractive new effect measure for clinical trials with composite endpoints.

## Additional files

Additional file 1 The additional file contains further simulation results with the distributions described in this work but other weighting schemes.(PDF 172 kb)

## Data Availability

Simulated data and R programs can be obtained from the authors upon request.
