# Supplementary material for: Introducing a new estimator and test for the weighted all-cause hazard ratio
Source: BMC Med Res Methodol. 2019 Jun 11;19:118. doi: 10.1186/s12874-019-0765-1 (PMC6560733; doi:10.1186/s12874-019-0765-1)
Supplement: Supplementary file 1 — The additional file contains further simulation results with the distributions described in this work but other weighting schemes.(PDF 172 kb) [file 12874_2019_765_MOESM1_ESM.pdf]

TECHNICAL ADVANCE

Additional file to the article 'Introducing a new estimator and test for the weighted all-cause hazard ratio': Further Simulation Results

Ann-Kathrin Ozga* and Geraldine Rauch†

*Correspondence: a.ozga@uke.de
Institute of Medical Biometry and Epidemiology, University Medical Center Hamburg-Eppendorf, Martinistraße 52, 20246 Hamburg, Germany
Full list of author information is available at the end of the article
†geraldine.rauch@charite.de

Additional Simulation Results

The following two tables show further simulation results with weights of 1 and 0.7 and the same distributions as considered in the main manuscript. The number of simulations is also 1000 with 1000 runs in the permutation test. A total of 100 individuals per treatment group are considered with a minimal follow-up of 1 or 2 years.

Table 1 Simulation results.

Sc.	Assumptions for original parametric estimator*	Assumptions for new non-parametric estimator#	τ	Weights		Ln of True WHR	Mean number of events (sd)		Mean of estimated Ln(WHR) (sd)		Power for permutation test	Power for weight-based log-rank test
				w_{EP_1}	w_{EP_2}	$ln(\theta^w_{CE}(\tau))$	EP_1	EP_2	$ln(\hat{\theta}^w_{CE}(\tau))$	$ln(\tilde{\theta}^w_{CE}(\tau))$	$\hat{\theta}^w_{CE}(\tau)$	$\tilde{\theta}^w_{CE}(\tau)$
**1a**	✓	✗	1	1	0.7	-0.86	50.21 (6.51)	35.16 (5.21)	-0.87 (0.25)	-0.75 (0.24)	0.97	0.97
**1b**			2	1	0.7	-0.97	72.60 (6.74)	80.13 (6.73)	-0.98 (0.17)	-0.86 (0.17)	1.00	1.00
**1c**			2	0.7	1	-1.07			-1.08 (0.19)	-0.98 (0.17)	1.00	1.00
**2a**	✗	✗	1	1	0.7	-0.70	48.22 (6.08)	37.15 (5.33)	-0.77 (0.25)	-0.76 (0.24)	0.92	0.98
**2b**			2	1	0.7	-0.68	67.79 (6.75)	51.93 (5.60)	-0.75 (0.20)	-0.73 (0.19)	0.98	0.99
**3a**	✓	✗	1	1	0.7	-0.19	39.97 (5.63)	47.94 (5.87)	-0.20 (0.24)	-0.36 (0.22)	0.18	0.60
**3b**			1	0.7	1	0.07			0.05 (0.24)	-0.08 (0.22)	0.01	0.18
**3c**			2	1	0.7	0.06	69.84 (6.39)	124.63 (6.40)	0.06 (0.17)	-0.08 (0.17)	0.02	0.48
**4a**	✗	✗	1	1	0.7	-0.32	62.84 (6.39)	6.49 (2.50)	-0.14 (0.69)	-0.20 (0.25)	0.07	0.23
**4b**			1	0.7	1	-0.26			-0.08 (0.73)	-0.16 (0.25)	0.04	0.23
**4c**			2	1	0.7	-0.42	86.66 (6.95)	24.29 (4.57)	-0.20 (0.24)	-0.25 (0.19)	0.14	0.43
**4d**			2	0.7	1	-0.39			-0.17 (0.28)	-0.23 (0.20)	0.09	0.40
**5a**	✗	✗	1	1	0.7	-0.45	133.27 (6.26)	19.31 (4.05)	-0.65 (0.38)	-0.71 (0.16)	0.94	1.00
**5b**			1	0.7	1	-0.35			-0.51 (0.42)	-0.61 (0.17)	0.66	1.00
**6a**	✓	✗	2	1	0.7	0.00	67.04 (6.74)	56.49 (6.37)	0.00 (0.18)	0.00 (0.18)	0.02	0.07
**6b**			2	0.7	1	0.60			0.60 (0.16)	0.60 (0.16)	0.00	0.06
**7a**	✓	✓	2	1	0.7	0.51	15.83 (3.84)	141.89 (6.18)	0.51 (0.17)	0.52 (0.16)	0.00	0.00
**7b**			2	0.7	1	0.15			0.16 (0.18)	0.16 (0.21)	0.01	0.00
**8a**	✗	✓	2	1	0.7	-0.74	108.25 (6.93)	78.79 (6.69)	-0.90 (0.19)	-0.75 (0.16)	1.00	1.00
**8b**			2	0.7	1	-0.86			-1.02 (0.20)	-0.87 (0.16)	1.00	1.00
**9a**	✗	✗	1	0.7	1	-0.61	132.78 (6.43)	15.22 (3.56)	-0.76 (0.21)	-0.63 (0.16)	0.97	0.99
**9b**			2	1	0.7	-0.53	178.24 (4.21)	21.76 (4.21)	-0.85 (0.27)	-0.58 (0.16)	0.99	0.99
**9c**			2	0.7	1	-0.55			-0.97 (0.33)	-0.63 (0.18)	0.98	1.00
**10a**	✗	✗	1	1	0.7	-0.74	84.95 (7.02)	62.44 (6.34)	-0.73 (0.17)	-0.74 (0.17)	0.99	1.00
**10b**			1	0.7	1	-0.80			-0.82 (0.17)	-0.84 (0.17)	1.00	1.00
**10c**			2	1	0.7	-0.74	113.64 (6.92)	80.58 (6.55)	-0.68 (0.14)	-0.75 (0.16)	1.00	1.00
**10d**			2	0.7	1	-0.76			-0.76 (0.14)	-0.83 (0.16)	1.00	1.00

Ln: natural logarithm; WHR: Weighted all-cause hazard ratio; sd: Standard deviation;

*It is assumed that the Weibull model used to estimate the cause-specific hazards is the correct one;

#It is assumed that the cause-specific baseline hazards are equal.

Table 2 Simulation results: Performance.

Sc.	Amount of Simulations		Bias		Standardized Bias		$\sqrt{\text{Mean Square Error}}$		Relative Efficiency	Coverage*	
	$ln(\hat{\theta}^w_{CE}(\tau))$	$ln(\tilde{\theta}^w_{CE}(\tau))$	$ln(\hat{\theta}^w_{CE}(\tau))$	$ln(\tilde{\theta}^w_{CE}(\tau))$	$ln(\hat{\theta}^w_{CE}(\tau))$	$ln(\tilde{\theta}^w_{CE}(\tau))$	$ln(\hat{\theta}^w_{CE}(\tau))$	$ln(\tilde{\theta}^w_{CE}(\tau))$	$\frac{MSE(ln(\hat{\theta}^w_{CE}(\tau)))}{MSE(ln(\tilde{\theta}^w_{CE}(\tau)))}$	$ln(\hat{\theta}^w_{CE}(\tau))$	$ln(\tilde{\theta}^w_{CE}(\tau))$
**1a**	991	1000	-0.02	0.10	-0.07	0.43	0.25	0.26	0.93	92.84	90.00
**1b**	997	1000	-0.01	0.11	-0.05	0.66	0.17	0.20	0.75	94.48	88.80
**1c**	997	1000	-0.01	0.09	-0.05	0.50	0.19	0.20	0.97	95.29	92.30
**2a**	1000	1000	-0.07	-0.06	-0.30	-0.26	0.26	0.25	1.08	92.70	92.50
**2b**	999	1000	-0.07	-0.06	-0.36	-0.31	0.21	0.20	1.09	91.89	93.10
**3a**	1000	1000	-0.02	-0.17	-0.06	-0.78	0.24	0.28	0.73	92.60	87.30
**3b**	1000	1000	-0.02	-0.15	-0.06	-0.67	0.25	0.27	0.85	93.60	89.40
**3c**	1000	1000	0.00	-0.14	-0.01	-0.82	0.17	0.22	0.60	92.48	78.95
**4a**	993	1000	0.17	0.12	0.25	0.47	0.72	0.28	6.77	90.76	91.94
**4b**	993	1000	0.18	0.09	0.24	0.36	0.76	0.26	8.13	92.56	92.65
**4c**	996	1000	0.21	0.17	0.90	0.85	0.32	0.26	1.53	84.21	85.57
**4d**	996	1000	0.22	0.17	0.78	0.84	0.36	0.26	1.88	87.42	86.17
**5a**	995	1000	-0.21	-0.26	-0.55	-1.62	0.43	0.31	1.91	82.50	62.56
**5b**	995	1000	-0.16	-0.26	-0.39	-1.55	0.45	0.31	2.08	89.74	64.56
**6a**	998	1000	0.00	0.00	0.00	0.00	0.18	0.18	1.04	95.19	95.20
**6b**	998	1000	-0.00	-0.00	-0.01	-0.00	0.20	0.18	1.26	95.89	95.40
**7a**	984	1000	0.00	0.01	0.00	0.04	0.17	0.16	1.08	94.51	94.09
**7b**	984	1000	0.00	0.01	0.01	0.04	0.16	0.16	1.01	95.41	94.89
**8a**	1000	1000	-0.16	-0.01	-0.83	-0.03	0.25	0.16	2.45	84.20	93.30
**8b**	1000	1000	-0.17	-0.01	-0.84	-0.04	0.26	0.16	2.56	83.80	92.90
**9a**	998	998	-0.15	-0.02	-0.75	-0.13	0.26	0.17	2.42	88.68	95.35
**9b**	990	1000	-0.32	-0.05	-1.19	-0.28	0.41	0.17	6.25	64.14	92.20
**9c**	990	1000	-0.42	-0.08	-1.30	-0.49	0.53	0.19	7.60	55.86	88.30
**10a**	1000	1000	0.01	-0.00	0.06	-0.02	0.17	0.17	0.98	94.30	94.70
**10b**	1000	1000	-0.02	-0.03	-0.11	-0.20	0.17	0.17	0.97	93.90	94.20
**10c**	1000	1000	0.06	-0.01	0.45	-0.05	0.16	0.16	0.95	92.40	92.70
**10d**	1000	1000	-0.01	-0.08	-0.03	-0.49	0.14	0.18	0.62	94.20	90.10

* Defined as proportion of times the 95%-confidence interval for the estimator includes the true effect.
